# A validated Screening instrument for Child Abuse and Neglect (SCAN) at the emergency department

**DOI:** 10.1007/s00431-022-04635-0

**Published:** 2022-10-05

**Authors:** F. Hoedeman, P. J. Puiman, E. A. L. van den Heuvel, M. J. Affourtit, R. Bakx, M. W. Langendam, E. M. van de Putte, I. M. B. Russel-Kampschoer, M. C. M. Schouten, A. H. Teeuw, H. J. de Koning, H. A. Moll

**Affiliations:** 1 Department of General Paediatrics, Erasmus University Medical Centre Sophia Children’s Hospital, P.O. Box 2060, 3000 CB Rotterdam, the Netherlands; 2 Department of Paediatrics, University Medical Centre Utrecht Wilhelmina Children’s Hospital, Utrecht, the Netherlands; 3 Department of Paediatric Surgery, Amsterdam University Medical Centre Emma Children’s Hospital, Amsterdam, the Netherlands; 4 Department of Epidemiology and Data Science, Amsterdam Public Health Institute, Amsterdam University Medical Centre, Amsterdam, the Netherlands; 5 Department of Paediatrics, University Medical Centre Groningen Beatrix Children’s Hospital, Groningen, the Netherlands; 6 Department of Social Paediatrics, Amsterdam University Medical Centre Emma Children’s Hospital, Amsterdam, the Netherlands; 7 Department of Public Health, Erasmus University Medical Centre, Rotterdam, the Netherlands

**Keywords:** Child maltreatment, Emergency medicine, Screening, Checklist

## Abstract

The objective of this study is to develop and validate a screening instrument for the recognition of child maltreatment in the emergency department (ED). Existing data on screening questions and outcomes (diagnosis of child maltreatment) from three large observational screening studies at eight different EDs in the Netherlands were harmonized. A multivariate logistic regression was performed to develop the Screening instrument for Child Abuse and Neglect (SCAN). The SCAN was validated by performing a cross-validation and calculating the discriminative ability. A total of 24,963 patients were included. Out of the potential screening questions the following questions were included in the final instrument: is the injury compatible with the history, and does it correspond to the child’s developmental level? (aOR 10.40, 95% CI 5.69–19.02), was there an unnecessary delay in seeking medical help? (aOR 3.45, 95% CI 1.73–6.88) and is the behaviour/interaction of the child and parents (carers) appropriate? (aOR 14.67, 95% CI 7.93–27.13). The SCAN had a pooled AUC of 0.75 (95% CI 0.63–0.87) in the cross-validation. The question “Are there other signals that make you doubt the safety of the child and/or family?” (available in only one of the original datasets, OR 182.9; 95% CI 102.3–327.4) was by consensus added to the final SCAN.

*Conclusion*: This validated and brief Screening instrument for Child Abuse and Neglect (SCAN) is designed to improve early recognition of child maltreatment in the ED. A positive screening result of the SCAN warrants a thorough work-up for child maltreatment, including a top-toe examination, if necessary additional diagnostics and adequate safety measures.

**Table:** 

**What is Known:** *• Screening instruments increase the detection of possible cases of child maltreatment in the emergency department and support health care professionals to identify these cases.* *• The application of different screening instruments led to the need for one brief validated instrument.*	
**What is New:** *• This study presents a validated and brief Screening instrument for Child Abuse and Neglect (SCAN), consisting of four questions.* *• The SCAN supports professionals in detecting signals of child maltreatment, and a positive screening result should lead to a thorough work-up, including a top-toe examination, complete history, additional diagnostic tests and consultation of a child abuse expert.*

**Supplementary Information:**

The online version contains supplementary material available at 10.1007/s00431-022-04635-0.

## Introduction

In Europe, at least 850 children under 15 years of age die from child maltreatment annually, a mortality rate of 0.54 per 100,000, with higher death rates in children under 5 years (0.78 per 100,000) [1, 2]. Since child maltreatment remains difficult to identify, the number of child maltreatment victims is most likely to be underestimated, especially during the recent COVID-19 pandemic [1, 3–5]. Early recognition and subsequent interventions are crucial, as child maltreatment (sexual, physical and emotional abuse, and neglect) has a negative impact on children’s lifelong physical and mental health, and entails a huge socioeconomic burden [6–9].

Emergency departments (EDs) are the gateway to acute health care and are important for the early detection of child maltreatment [10, 11]. Different screening tools showed improvement in the detection of child maltreatment at the ED [11–16]. In a survey amongst ED professionals in Europe, only 29% of respondents used a checklist for the detection of child maltreatment [17]. Of these, 32% used a validated instrument based on the SPUTOVAMO or ESCAPE screening tools and 68% used a local screening tool [17]. Screening instruments such as the ESCAPE and SPUTOVAMO have been validated to identify suspected cases of child maltreatment in the ED [12–15, 18]. The discriminative value of local screening instruments is unknown.

At an ED, screening instruments need to be short and feasible. A full top-toe examination as part of standard screening has led to a low adherence since ED professionals often experience a lack of time and reluctance to completely undress a child [15, 19, 20]. Teeuw et al. showed that in only 34% of the children, a top-toe examination was performed when combined with the SPUTOVAMO screening checklist [19].

The availability of several screening tools with variability in questions and validity, and low adherence due to a top-toe examination for each child, calls for one effective screening instrument. Such an instrument needs to be brief and attainable to ensure screening in the ED with maximal adherence of ED staff. Hence, we aimed to develop a validated screening instrument for the recognition of (suspected) Child Abuse and Neglect (SCAN) in the ED based on the best discriminative questions from three previous validation studies.

## Methods

### Study design

A National Child Abuse Consortium (NCAC) was established to develop, validate and implement the SCAN based on existing databases from three observational studies at EDs in the Netherlands:

1. Amsterdam University Medical Centre, located in the Academic Medical Centre (AMC), developed and tested the SPUTOVAMO 9-item questionnaire and separately tested the top-toe examination for its accuracy in detecting child maltreatment (for details see Teeuw et al. [15]). The study population consisted of all children under 18 years who presented at the ED between 1 January 2011 and 1 July 2013, of which 46% (7988/17,229) were screened (Online Resource 1).
2. Erasmus University Medical Centre (EMC) developed and tested the ESCAPE 6-item instrument which was implemented in one university and two teaching hospital EDs in the Rotterdam region (for details see Louwers et al. [13, 21]). The study population consisted of all children aged 0 to 18 years visiting the EDs between February 2008 and December 2009, of which 48% (18,275/38,136) were screened (Online Resource 1).
3. University Medical Centre Utrecht (UMC) developed and tested the SPUTOVAMO-R 6-item questionnaire [18]. One university and three teaching hospital EDs in the Utrecht region participated (for details see Sittig et al. [18]). The study population consisted of all children aged 0 to 7 years, who were referred to the ED because of a physical injury between June 2009 and December 2010, of which all 4290 children were screened with the SPUTOVAMO-R (Online Resource 1).

From these three studies, all children who were presented and screened at the ED were included for the purpose of this study. The screening was performed by ED nurses (or physicians) during triage or directly after the clinical examination of the patients [13, 15, 18]. The use of screening tools and interpretation of the questions is always supported by training of ED staff including training on communication in case of suspicion of child maltreatment [13, 15]. All instruments were considered positive for (suspected) child maltreatment when at least one of the questions was aberrant (positive for possible maltreatment). When a suspicion of child maltreatment arose at the ED, based on a positive screening result, the hospital child abuse team was consulted. The local multidisciplinary child abuse team of the hospitals from all three studies evaluated all positive screened cases [15, 18, 21] and subsequently assessed if and which actions needed to be taken. To ensure health care professionals take the appropriate actions on reporting cases with suspected child maltreatment, there is a step-by-step ‘child abuse report’ guideline in the Netherlands [22]. This guideline includes consultation of and/or referral to the regional Safe at Home centre. These centres form a national network and are tasked with investigating suspected child maltreatment cases, assessing the need for help and, if necessary, reporting to child protective services [23].

### Harmonization of data

The different questions of the three instruments were harmonized by linking questions with the same meaning in their original language (Dutch) together with the project leaders of the original studies and the NCAC (EP, HK, HM, IR, MA, ML, MS, PP, RB and RT) (Online Resource 2).

Question 3 (consistent history) was not present in the SPUTOVAMO questionnaire from the AMC dataset. Question 6 (doubts about the safety of the child/family) was only present in the ESCAPE instrument from the EMC dataset. However, since this question had a relatively high odds ratio (OR 182.9; 95% CI 102.3–327.4) in the EMC study and it might also indicate neglect or emotional abuse, the consortium decided to add this question to the SCAN [21]. Questions with the best predictive value were included in the final screening instrument (SCAN).

The reference standard for the AMC and EMC studies was defined as the consensus diagnosis of (suspected) child maltreatment by the local multidisciplinary child abuse team. The reference standard for the UMC study was defined as physical child abuse and/or neglect based on the decision of at least two out of three experts, blinded for their mutual decision [18]. The consensus diagnosis for child maltreatment, decided by the multidisciplinary child abuse team or expert team independent of the screening result, was the primary outcome.

Only a small proportion of the children with a negative screening result received a multidisciplinary consensus diagnosis for (suspected) child maltreatment (*n* = 693). Thus, we defined two outcome measures:

(A) Positive consensus diagnosis for child maltreatment for the positive screened (*n* = 868) and for the negative-screened cases (*n* = 24,095). For the negative-screened children who received no evaluation by the multidisciplinary child abuse or expert team and therefore had no consensus diagnosis (*n* = 23,402), the outcome was considered no child maltreatment.
(B) Positive consensus diagnosis for child maltreatment for the positive-screened and for the negative-screened cases with the addition of the regional Safe at Home centre reports for the negative-screened cases. The consortium decided a report to the regional Safe at Home centre was the closest to the consensus diagnosis of the hospital child abuse team. Therefore, all negative-screened children were monitored for reports to the regional Safe at Home centre up to 3 (EMC) to 6 months (AMC and UMC) after the ED visit [24]. Based on a previous study, a period of 6 months would be sufficient to determine child maltreatment related to the presentation at the ED [15]. For the negative-screened cases, a report to the regional child abuse center was defined as a positive consensus diagnosis and was added as an outcome. If no report was filed, the outcome was assumed to be negative.

### Statistical analysis

Descriptive and univariate analyses of the different questions in each dataset were performed with the primary outcome. Cases with missing answers from the original datasets for two or more of the harmonized questions were excluded from analyses. The three different databases (AMC, EMC and UMC) were combined into one composite database. Question 3 regarding consistent history in the AMC dataset and any remaining missing values on the harmonized questions in all datasets were imputed. The model for multiple imputation included the following constraints: dataset, hospital, gender, age, consensus diagnosis (outcome measure A) and all harmonized questions. Based on the amount of missing values, a total of *n* = 10 imputation sets were used and results (regression coefficients, *p*-values and aORs) on imputed data were pooled according to Rubin’s rules [25, 26]. A sensitivity analysis was performed based on the estimate that missing values on the harmonized questions were negative.

Multivariate logistic regression was performed to derive a final screening instrument with the harmonized questions as variables for both outcome measures. The model was adjusted for age, gender, hospital (*n* = 8) and all other harmonized questions.

After the determination of the final set of questions (SCAN), this model was validated by internal–external cross-validation. We derived the screening instrument on two datasets, validated it in the third dataset and repeated this analysis three times, in different derivation and validation sets. Whereas classic validation splits the data into random derivation and validation sets to assess the internal validity of the model, the internal–external validation method uses all available data to develop the model and uses cross-validation to validate the model three times [27, 28].

To assess whether the SCAN performed similar for children under 5 years, who are at higher risk for child maltreatment [1, 14], a subgroup analysis with only the patients under 5 years old was performed.

The discriminative ability of the model was evaluated with the area under the curve (AUC, interpretation: low (0.5 ≥ AUC ≤ 0.7), moderate (0.7 ≥ AUC ≤ 0.9) or high (0.9 ≥ AUC ≤ 1.0)) [29]. Calibration of the model was assessed by calibration plots with observed outcomes versus expected probabilities.

Calibration was performed with the R version 4.0.2 software package. For all other analyses, we used SPSS version 28.

## Results

A total of 30,553 patients were screened for child maltreatment (Online Resource 1). We excluded 4972 (16.3%) cases with missing answers to two or more questions and 618 (2.0%) cases with missing gender, resulting in 24,963 eligible cases for analysis (Online Resource 3). The median age was 3.8 years (IQR 1.4–7.7), 56.7% were male and 102 (0.4%) had a positive outcome on the consensus diagnosis (outcome measure A) (Table 1).

**Table 1:** Baseline characteristics of the included cases

Characteristics	Dataset AMC *n* = 3136 (%)	Dataset EMC *n* = 18,159 (%)	Dataset UMC *n* = 3668 (%)	Total dataset *n* = 24,963 (%)
Gender				
Boy	1753 (55.9)	10,266 (56.5)	2138 (58.3)	14,157 (56.7)
Girl	1383 (44.1)	7893 (43.5)	1530 (41.7)	10,806 (43.3)
Age median (IQR)	6.2 (2.4–11.6)	3.3 (1.1–8.2)	4.0 (2.5–5.5)	3.8 (1.4–7.7)
< 4 years	1154 (36.8)	9985 (55.0)	1821 (49.6)	12,960 (51.9)
≥ 4 to < 8 years	628 (20.0)	3522 (19.4)	1847 (50.4)	5997 (24.0)
≥ 8 to < 12 years	649 (20.7)	2592 (14.3)	n.a	3241 (13.0)
≥ 12 to < 19 years	705 (22.5)	2060 (11.3)	n.a	2765 (11.1)
Positive screening result before multiple imputation of screening questions*				
Yes	267 (8.5)	403 (2.2)	121 (3.3)	791 (3.2)
Positive screening result after multiple imputation of screening questions*				
Yes	337 (10.7)	407 (2.2)	124 (3.4)	868 (3.5)
Outcome measure A (consensus diagnosis)				
Yes	41 (1.3)	52 (0.3)	9 (0.2)	102 (0.4)
Outcome measure B (consensus diagnosis & reports Safe at Home centre for screened negatives)				
Yes	90 (2.9)	65 (0.4)	54 (1.5)	209 (0.8)

*n.a.* not applicable

^*^See Table 2 for screening questions before multiple imputation, and Online Resource 5 for screening questions after multiple imputation

Predictive values of the original questions in each dataset were calculated (Online Resource 4), and hereafter the questions were harmonized (Online Resource 2). Table 2 and Online Resource 5 show the distribution of the harmonized questions respectively before and after multiple imputation of missing values.

**Table 2:** Screening questions

Harmonized screening questions	Dataset AMC *n* = 3136 (%)	Dataset EMC *n* = 18,159 (%)	Dataset UMC *n* = 3668 (%)	Total dataset *n* = 24,963 (%)
Question 1: injury compatible with history and corresponding to child’s developmental level				
Yes	2004 (63.9)	17,039 (93.8)	3606 (98.3)	22,649 (90.7)
No*	127 (4.0)	79 (0.4)	62 (1.7)	268 (1.1)
Missing	1005 (32.0)	1041 (5.7)	0 (0)	2046 (8.2)
Question 2: unnecessary delay in seeking medical help				
Yes*	77 (2.5)	137 (0.8)	40 (1.1)	254 (1.0)
No	2930 (93.4)	17,952 (98.9)	3628 (98.9)	24,510 (98.2)
Missing	129 (4.1)	70 (0.4)	0 (0)	199 (0.8)
Question 3: consistent history				
Yes	n.a	18,059 (99.4)	3648 (99.5)	21,707 (87.0)
No*	n.a	78 (0.4)	19 (0.5)	97 (0.4)
Missing	3136 (100)	22 (0.1)	1 (0.0)	3159 (12.7)
Question 4: appropriate behaviour of the child, the parents and appropriate interaction				
Yes	3076 (98.1)	18,012 (99.2)	3646 (99.4)	24,734 (99.1)
No*	60 (1.9)	83 (0.5)	21 (0.6)	164 (0.7)
Missing	0 (0)	64 (0.4)	1 (0.0)	65 (0.3)
Question 5: physical injuries found with top-toe examination suspect for child maltreatment				
Yes*	69 (2.2)	52 (0.3)	31 (0.8)	152 (0.6)
No	3067 (97.8)	18,059 (99.4)	3451 (94.1)	24,577 (98.5)
Missing	0 (0)	48 (0.3)	186 (5.1)	234 (0.9)
Question 6: other signals that make you doubt the safety of child/family				
Yes*	n.a	163 (0.9)	n.a	n.a
No	n.a	17,878 (98.5)	n.a	n.a
Missing	n.a	118 (0.6)	n.a	n.a

*n.a.* not applicable

^*^positive answer on screening question = positive result. See Online Resource 5 for screening questions after multiple imputation

In the multivariate logistic regression analysis with outcome measure A, the complete model showed increased aORs for questions 1, 2, 4 and 5 (Table 3). For outcome measure B, these aORs show comparable results to outcome measure A.

**Table 3:** Complete and reduced adjusted model for the consensus diagnosis of child maltreatment (outcome measure A and B)

**Complete model**		**Outcome measure A**		**Outcome measure B**	
		aOR (95% CI)	*p*-value	aOR (95% CI)	*p*-value
(Constant)		0.005 (0.003–0.010)	0.98	0.015 (0.010–0.021	0.000
Question 1	Injury compatible with history and corresponding to child’s developmental level	8.35 (4.33–16.11)	< 0.001	4.39 (2.54–7.60)	< 0.001
Question 2	Unnecessary delay in seeking medical help	2.31 (1.07–5.00)	0.03	1.71 (0.85–3.44)	0.13
Question 3	Consistent history	0.99 (0.30–3.26)	0.99	1.61 (0.56–4.66)	0.38
Question 4	Appropriate behaviour of the child, the parents and appropriate interaction	6.91 (3.41–14.02)	< 0.001	5.27 (2.80–9.94)	< 0.001
Question 5	Physical injuries found with top-toe examination suspect for child maltreatment	7.62 (3.69–15.75)	< 0.001	5.06 (2.66–9.63)	< 0.001

**Reduced model**		**Outcome measure A**		**Outcome measure B**	
		aOR (95% CI)	*p*-value	aOR (95% CI)	*p*-value
(Constant)		0.006 (0.003–0.01)	0.98	0.015 (0.010–0.021)	0.000
Question 1	Injury compatible with history and corresponding to child’s developmental level	10.40 (5.69–19.02)	< 0.001	5.54 (3.37–9.13)	< 0.001
Question 2	Unnecessary delay in seeking medical help	3.45 (1.73–6.88)	< 0.001	2.46 (1.32–4.57)	0.005
Question 4	Appropriate behaviour of the child, the parents and appropriate interaction	14.67 (7.93–27.13)	0.000	10.59 (6.21–18.07)	0.000

Models are adjusted for age, gender, hospital (*n* = 8) and the screening questions. Outcome measure A: unknown outcomes of screened negatives were analysed as a negative outcome. Outcome measure B: unknown outcomes for the negative-screened cases were supplemented based on the reports to the Safe at Home centre

The complete model with outcome measure A had an AUC of 0.80 (95% CI 0.74–0.87). In a reduced model, question 3 regarding a consisting history was left out since the question was not significant in the model and the aim was to develop a brief instrument. In line with this aim and to assure maximal adherence from ED staff, question 5 regarding the top-toe examination was also excluded after showing to be non-contributive to the model performance (Table 3). This reduced model consisting of 3 questions discriminated at a level comparable (AUC 0.79, 95% CI 0.73–0.85) to the complete model including all 5 questions. In the sensitivity analysis, the reduced model provided similar coefficients and model performance when all missing values on the screening questions in the original datasets were assumed to be negative answers (Online Resource 6). With outcome measure B, the complete and reduced model discriminated moderately to poorly (resp. AUC 0.66, 95% CI 0.61–0.70 and AUC 0.64, 95% CI 0.59–0.68).

**Fig. 1:**
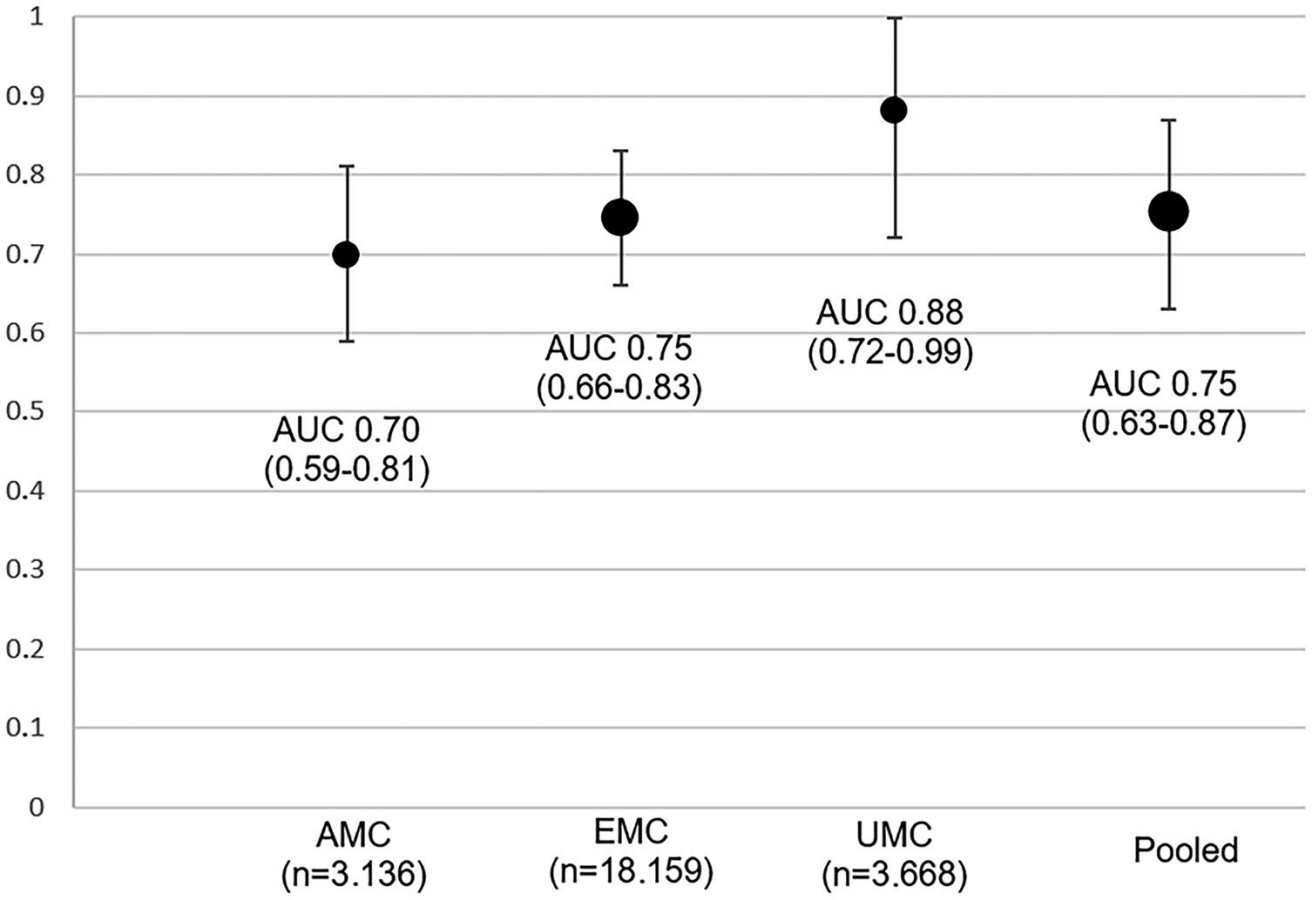
Discriminative value (AUC with 95% confidence intervals) of the reduced model for the consensus diagnosis of child maltreatment for 3 internal–external cross-validations. Unknown outcomes of the screened negatives are analysed as negative outcome (outcome measure A)

In the cross-validation, the pooled AUC of the reduced model was 0.75 (95% CI 0.63–0.87) for outcome measure A and 0.61 (95% CI 0.54–0.68) for outcome measure B (Fig. 1). Calibration was poor for the different internal–external validations with outcome measure B (range slope 0.55 to 1.22, range intercept −1.23 to −0.44) (Online Resource 7).

A total of 14,978 children (60.0%) were aged under 5 years old, of which 57.7% were male and 63 (0.4%) had a positive consensus diagnosis (outcome measure A). In the subgroup analysis of children aged under 5 years old, all models (reduced model and cross-validation of the reduced model) performed comparable to the models of the total group of children (*n* = 24,963) (Table 4).

**Table 4:** Model performance subgroup analysis children < 5 years old compared to all children

	**All children** **(*n* = 24,963)**	**Children < 5 years old** **(*n* = 14,978)**
**Reduced model**	**AUC (95%CI)**	**AUC (95%CI)**
Outcome measure A	0.79 (0.73–0.85)	0.79 (0.71–0.86)
Outcome measure B	0.64 (0.59–0.68)	0.64 (0.58–0.69)
**Cross-validation reduced model**	**Pooled AUC (95%CI)**	**Pooled AUC (95%CI)**
Outcome measure A	0.75 (0.63–0.87)	0.77 (0.62–0.93)
Outcome measure B	0.61 (0.54–0.68)	0.61 (0.51–0.71)

Outcome measure A: unknown outcomes of screened negatives were analysed as negative outcome. Outcome measure B: unknown outcomes for the negative-screened cases were supplemented based on the reports to the Safe at Home centre

*AUC* area under the curve

## Discussion

**Fig. 2:**
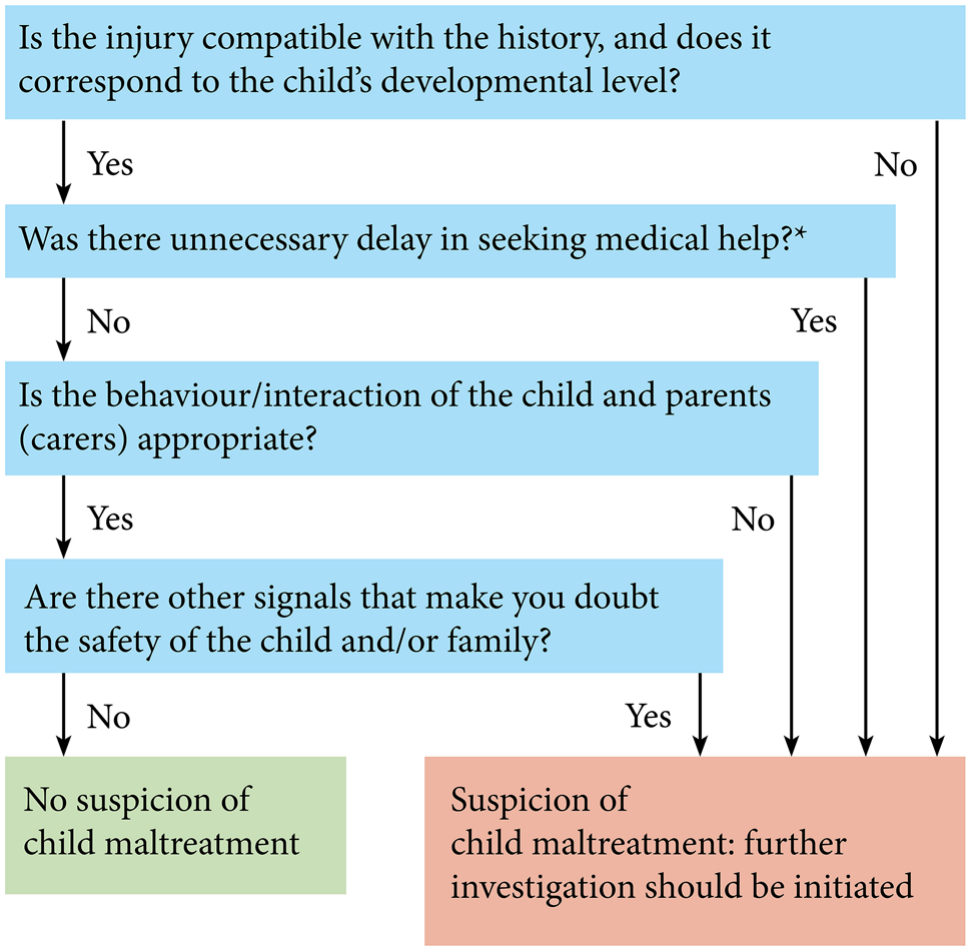
Screening instrument for Child Abuse and Neglect (SCAN). *only regarding the patient delay, no doctors delay

The SCAN is a brief screening questionnaire for child maltreatment developed and validated based on three observational studies from eight different emergency departments including nearly 25,000 children (Fig. 2). The SCAN showed a moderate performance and performed comparable in children under 5 years.

Previous studies have shown the need to support health care professionals in detecting signs of possible child maltreatment and the added value of using a screening instrument [16, 20, 30]. Louwers et al. showed that the detection rate for child maltreatment was significantly higher in children who were screened than in those who were not screened (0.5% vs. 0.1%, *p* < 0.001) [13]. Different screening tools (SPUTOVAMO, ESCAPE and local tools) are used at EDs in Europe, and 67.9% is local non-validated tools [17]. Studies assessing the diagnostic value of these tools all showed high negative predictive values but low-to-moderate positive predictive values for child maltreatment (Online Resource 8) [12, 15, 18, 21, 31]. Not one instrument performed outstandingly in detecting child maltreatment (Online Resource 8) [12, 15, 18, 21, 31].

The SCAN is intended to be the first signal for possible child maltreatment, completed by the ED professional at triage, and therefore, it needs to be possible to complete the instrument quickly. A survey amongst EDs in Europe showed most respondents did not use a screening tool [17], even though validated screening tools such as the ESCAPE instrument and SPUTOVAMO are available [18, 21]. One explanation could be that screening tools are often too time-consuming [19, 20], implying that any redundant questions should be avoided. The shorter the screening instrument, the more feasible it is to implement in a busy ED. Additionally, although the combination of a complete physical examination with the SPUTOVAMO checklist led to the detection of more (possible) child maltreatment cases, adherence to the protocol was modest [15, 19]. By crucial appraisal of the added value of the different questions of the validated ESCAPE and SPUTOVAMO screening tools and by excluding a top-toe examination, we aimed to develop such a brief instrument that resulted in the SCAN.

Appraisal of the ESCAPE and SPUTOVAMO screening questions showed no added value (low performance) of the question regarding the consistent history and therefore led to the exclusion of this question. The question regarding concerns for the safety of the child/family from the validated ESCAPE instrument was included in the SCAN by consensus of the consortium. Although we aimed to develop a brief instrument, this question covers more subtle signals for child maltreatment such as neglect or emotional abuse that are difficult to identify.

A positive screening result of the SCAN should lead to a thorough work-up for child maltreatment and should include a complete history, top-toe examination, additional diagnostic tests and consultation of a child abuse expert. It has already been shown that completion of a screening instrument resulted in a higher chance of having a top-toe examination performed (RR 5.4, 95% CI 3.8–7.5, *p* = 0.000) [19]. Screening is just the first step in the recognition and management of child maltreatment. In suspect cases, a thorough medical work-up should be followed by psychosocial evaluation and warranting the safety of the child including reporting to the regional Safe at Home centre and/or child protective services [22].

The strength of this study is that a large population of children from eight EDs in the Netherlands was included. Furthermore, through cross-validation of our screening tool in three datasets, we improved the generalizability of the results.

This study has some limitations:

1. *Different datasets*: first, the UMC dataset included children from 0 to 7 years old with a physical injury (abuse or neglect), whereas the other two datasets included children aged 0 to 18 years old with all clinical presentations. This could have influenced our results; since physical injuries are more likely to trigger suspicion for abuse, the positive predictive value of screening tools for child maltreatment has shown to be higher in older age groups (12 years and older) [15], and self-disclosure is less likely for younger children [32]. However, a subgroup analysis of children aged under 5 years old, making the results of the different datasets more comparable, showed the similar model performance to the analysis of all children. Second, for missing values on the screening questions, we performed multiple imputation for which the sensitivity analysis showed similar results, indicating our results are plausible after imputation.
2. *Outcome*: compared to the consensus diagnosis by the child abuse team from the AMC and EMC study, the expert team of the UMC study has used stricter criteria for the consensus diagnosis, which might explain a lower number of child maltreatment cases (*n* = 9). Variability in screening rates at the ED between the three studies (for AMC and EMC 50% of the children and for UMC all children) might have influenced the number of positive cases. This could also be explained by the various study designs: different study populations, regions (i.e., socioeconomic status), comorbidities, training of staff and hospitals (academic versus regional). However, the association between screening questions and outcome is not expected to be different, only the generalizability of the results might be influenced.
  By assuming the outcome for the negative-screened children as “no child maltreatment” (A), we could have underestimated the true outcome. On the other hand, by supplementing these outcomes with a referral to the regional Safe at Home centre (B), this could have been an overestimation, as the referral might not have been related to the ED visit.
3. *Number of cases*: due to the low incidence of suspected child maltreatment, the performance of our instrument was evaluated in cross-validations with a lower number of cases than what is optimal for validation (100 cases) [33]. The poor calibration could be due to the overfitting of the model but could also be explained by the heterogeneity in the different datasets [34]. Although calibration was poor, the discrimination of the instrument was moderate in the cross-validations and future implementation will show the value of the SCAN.

Despite the undeniable differences between the studies, we managed to achieve a well-harmonized brief screening tool to improve the early detection of child maltreatment.

## Conclusion

This study presents a brief validated screening instrument (SCAN) consisting of four questions to improve early recognition of child maltreatment in the emergency department. The SCAN is not a diagnostic tool to detect proven child maltreatment, but it is an instrument to identify early signs of, or high-risk situations for, child maltreatment. A positive SCAN warrants a thorough work-up including a top-toe examination, complete history, additional diagnostic tests and consultation of a child abuse expert. Recognizing these signs and situations and creating awareness are important to start early interventions to help children, parents and families. Implementation of this instrument combined with adequate training and a clear hospital policy will be the next step in confirming the broad effectiveness and feasibility of the SCAN and its value for increasing the recognition of child maltreatment in the ED.

## Supplementary Information

Below is the link to the electronic supplementary material.

Supplementary file1 (DOCX 346 KB)

## Data Availability

Online resources are online available as supplementary material. All data relevant to the study are included in the article or uploaded as supplementary information. Original data are available from the corresponding author upon reasonable request.

## Abbreviations

AMC: Amsterdam University Medical Centre, location AMC (Academic Medical Centre)
AUC: Area under the curve
ED: Emergency department
EMC: Erasmus University Medical Centre
NCAC: National Child Abuse Consortium
SCAN: Screening instrument for Child Abuse and Neglect
UMC: University Medical Centre Utrecht
