# Supplementary material for: A validated Screening instrument for Child Abuse and Neglect (SCAN) at the emergency department
Source: Eur J Pediatr. 2022 Oct 5;182(1):79–87. doi: 10.1007/s00431-022-04635-0 (PMC9534589; doi:10.1007/s00431-022-04635-0)
Supplement: Supplementary file 1 — Supplementary file1 (DOCX 346 KB) [file 431_2022_4635_MOESM1_ESM.docx]

**Supplementary files ‘A Validated Screening instrument for Child Abuse and Neglect (SCAN) at the emergency department’**

**Article title**: A Validated Screening instrument for Child Abuse and Neglect (SCAN) at the emergency department

**Journal**: European Journal of Pediatrics

**Authors**: F. Hoedeman, P.J. Puiman, E.A.L. van den Heuvel, M.J. Affourtit, R. Bakx, M.W. Langendam, E.M. van de Putte, I.M.B. Russel-Kampschoer, M.C.M. Schouten, A.H. Teeuw, H.J. de Koning, H.A. Moll

**Corresponding author:**

Dr. Patrycja J. Puiman

Department of General Paediatrics, Erasmus MC Sophia Children’s Hospital, Rotterdam, the Netherlands

E-mail: [p.puiman@erasmusmc.nl](mailto:p.puiman@erasmusmc.nl)

**Table of content**

**Online Resource 1**: Baseline characteristics of the different datasets

**Online Resource 2**: Harmonization of different screening questions

**Online Resource 3**: Flowchart of the included cases from the three datasets for imputation

**Online Resource 4**: Univariate regression analyses of the different screening questions in the three datasets

- **Appendix 4A**: Univariate analysis of questions from AMC dataset (SPUTOVAMO)
- **Appendix 4B**: Univariate analysis of questions from EMC dataset (ESCAPE)
- **Appendix 4C**: Univariate analysis of questions from UMC dataset (SPUTOVAMO)

**Online Resource 5:** Screening questions after imputation

**Online Resource 6**: Sensitivity analysis for missing values on screening questions (outcome measure A)

**Online Resource 7**: Calibration plot: observed proportion vs. predicted probability of the screening instrument for 3 internal-external cross-validations (outcome measure B)

**Online Resource 8**: Validity of screening instruments for the recognition of (suspected) child maltreatment at the emergency department

**Online Resource 1: Baseline characteristics of the different datasets**

| **Baseline characteristics of the different datasets** | | | | |
| --- | --- | --- | --- | --- |
| Characteristics | | AMC (%) | EMC (%) | UMC (%) |
| Total population of children presenting at the emergency department | | 17229 | 38136 | 4290 |
| Total screened | | 7988 (46.4) | 18275 (47.9) | 4290 (100) |
| Eligible study patients | | 7988 (*<18* *years*) | 18275 (*≤18 years*) | 3668 (*0-7 years*) |
| Hospitals (n) | | 1 | 3 | 4 |
| Gender | |  |  |  |
|  | Boy | 4510 (56.5) | 10322 (56.5) | 2138 (85.9) |
|  | Girl | 3487 (43.5) | 7953 (43.5) | 1530 (35.7) |
| Age (median), IQR | | 6.6 (2.4–12.2)  *max. 18 years* | 3.3 (1.1–8.2)  *max. 18 years* | 4.0 (2.5–5.5)  *max. 7 years* |
| Study period | | 2011/2012/2013 | 2008/2009 | 2009/2010 |
| Positive screening result | | 279 (3.5) | 420 (2.3) | 123 (2.9) |
| Primary outcome (positive consensus diagnosis) | | 78 (0.9) | 55 (0.3) | 9 (0.2) |

**Online Resource 2: Harmonization of different screening questions**

| Harmonization of different screening questions | | | | | |
| --- | --- | --- | --- | --- | --- |
|  | Questions UMC dataset | Questions AMC dataset | Questions EMC dataset | Topic questions |  |
| Question 1 | Injury compatible with history and corresponding with age of child? (Q1) | Is this a normal place for this kind of injury? (Q2)  Does the injury look usual? (Q3)  Does the appearance of the injury fit with the stated age? (Q4)  Does the explanation fit with sort, place and appearance of the injury? (Q5) | Does the onset of the injury fit with the developmental level of the child? (Q3) | Injury compatible with history and corresponding to child’s developmental level |  |
| Question 2 | Delay in ER attendance without satisfactory explanation? (Q3) | Were the undertaken measures appropriate? (Q8) | Was there unnecessary delay in seeking medical help? (Q2) | Unnecessary delay in seeking medical help |  |
| Question 3 | History consistent when repeated? (Q2) | - | Is the history consistent? (Q1) | Consistent history |  |
| Question 4 | Father/mother and child: appropriate behaviour/interaction? (Q5) | Is there inappropriate behaviour observed during top-to-toe examination? / Is there an abnormal interaction between child and parents/carers? (part of top-to-toe examination) | Is the behaviour of the child/the carers and the interaction appropriate? (Q4) | Appropriate behaviour of the child, the parents and appropriate interaction |  |
| Question 5 | Top-to-toe examination: suspect? (Q6) | Are there any injuries observed during top-to-toe examination? (part of top-to-toe examination) | Are the findings of the top-to-toe examination in accordance with the history? (Q5) | Physical injuries found with top-toe examination suspect for child maltreatment |  |
| Question 6 | - | - | Are there any other signals that make you doubt the safety of the child or other family members? (Q6) | Other signals that make you doubt the safety of the child and/or family |  |

** The next questions were excluded from analysis as they are available in just one database and the question about who accompanied the child had no discriminative ability in previous studies: What type of injury? (AMC, Q1); Who caused the accident, is this person present in the ED? (AMC, Q6); Are the witnesses present in the ED? (AMC, Q7); Unexplained (other) injury in history? (UMC, Q4)***Online Resource 3: Flowchart of the included cases from the three datasets for imputation**

**
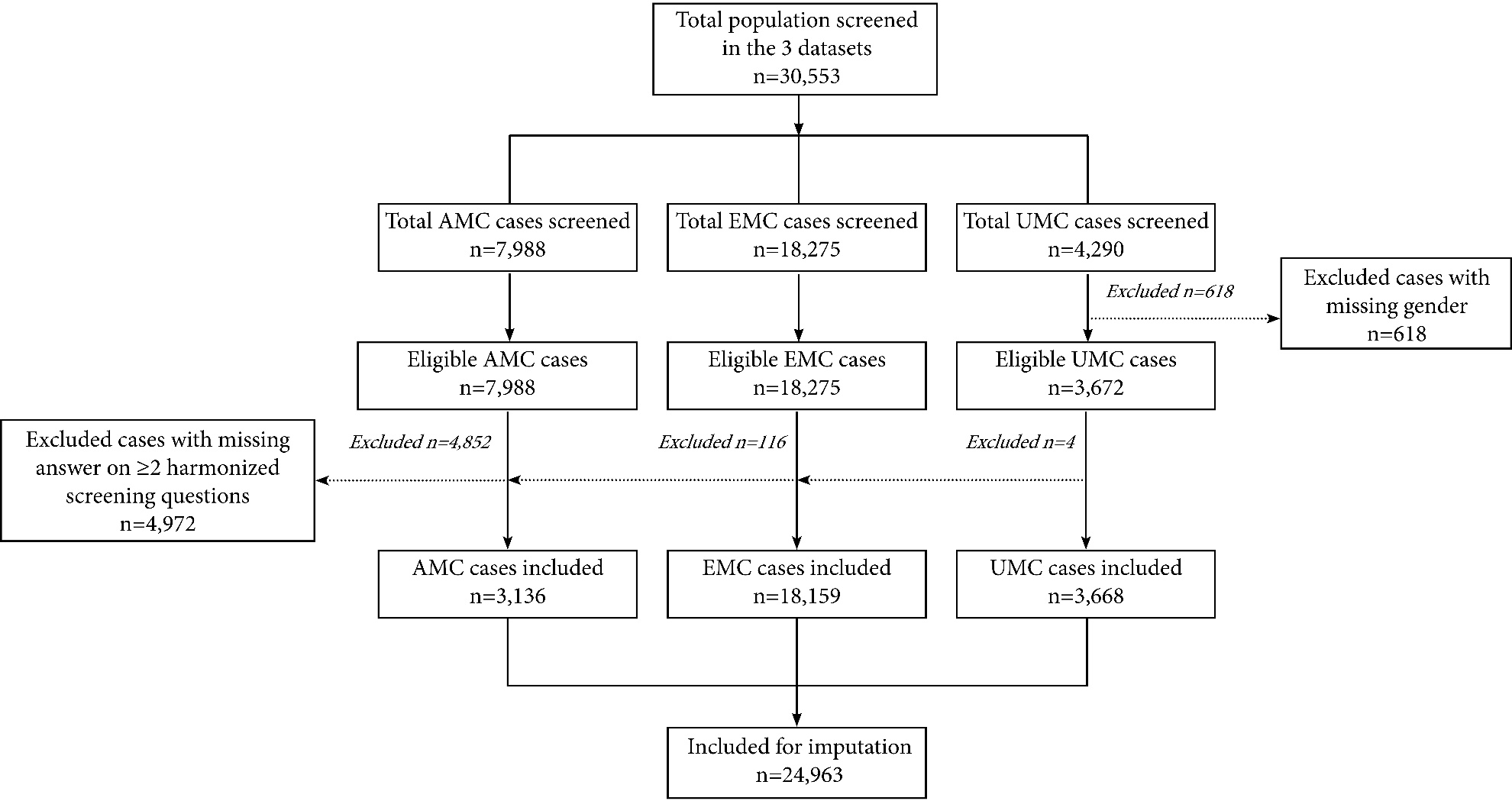
**

**Online Resource 4: Univariate regression analyses of the different screening questions in the three datasets**

**4A: Univariate analysis of questions from AMC dataset (SPUTOVAMO)**

| **Univariate analysis of questions from AMC dataset (SPUTOVAMO)** | | | | | | |
| --- | --- | --- | --- | --- | --- | --- |
| Items | Positive answers | No. of positive answers in total population n=7988 (%) | No. of positive answers among cases of suspected abuse (consensus diagnosis) n=78 (%) | OR (95% CI) | Sensitivity (95% CI) | Specificity (95% CI) |
| Is this a normal place for this kind of injury? | No | 102 (1.3) | 14 (17.9) | 20.3  (10.4–39.6) | 0.32 (0.19–0.48) | 0.98 (0.97–0.98) |
| Does the injury look usual? | No | 73 (0.9) | 10 (12.8) | 18.8  (8.8–40.1) | 0.25 (0.13–0.41) | 0.98 (0.98–0.99) |
| Does the appearance of the injury fit with the stated age? | No | 36 (0.5) | 4 (5.1) | 12.2  (4.1–36.2) | 0.10 (0.03–0.23) | 0.99* |
| Does the explanation fit with sort, place and appearance of the injury? | No | 111 (1.4) | 9 (11.5) | 9.9  (4.6–21.4) | 0.23 (0.11–0.38) | 0.97 (0.97–0.98) |
| Is there inappropriate behaviour observed during top-to-toe examination? / Is there an abnormal interaction between child and parents/carers? (part of top-to-toe examination) | Yes | 73 (0.9) | 13 (16.7) | 22.0  (11.1–43.5) | 0.26 (0.15–0.40) | 0.98 (0.98–0.99) |
| Are there any (physical) injuries observed during top-to-toe examination? (part of top-to-toe examination) | Yes | 79 (1.0) | 19 (24.4) | 37.7  (20.2–70.5) | 0.38 (0.25–0.53) | 0.98 (0.98–0.99) |
| Were the undertaken measures appropriate? | No | 140 (1.8) | 14 (17.9) | 16.8  (8.9–31.7) | 0.26 (0.15–0.40) | 0.98 (0.98–0.98) |
| ≥1 question positive |  | 279 (3.5) | 62 (79.5) | 75.3  (47.5–119.4) | 0.68 (0.57–0.78) | 0.97 (0.97–0.98) |

*P-values for all items <0.001. Unknown outcome of screened negatives are analysed as negative outcome.*

** Maximum range of 95% CI 0.98–0.99*

**4B: Univariate analysis of questions from EMC dataset (ESCAPE)**

| **Univariate analysis of questions from EMC dataset (ESCAPE)** | | | | | | | |
| --- | --- | --- | --- | --- | --- | --- | --- |
| Items | Positive answers | No. of positive answers in total population n=18275 (%) | No. of positive answers among cases of suspected abuse (consensus diagnosis) n=55 (%) | OR (95% CI) | Sensitivity (95% CI) | Specificity (95% CI) |  |
| Is the history consistent? | No | 83 (0.5) | 9 (16.4) | 50.0  (23.6–106.2) | 0.17 (0.08–0.30) | 0.99* |  |
| Was seeking medical help unnecessarily delayed? | Yes | 141 (0.8) | 6 (10.9) | 17.4  (7.3–41.3) | 0.12 (0.04–0.23) | 0.99* |  |
| Does the onset of the injury fit with the developmental level of the child? | No | 81 (0.4) | 17 (30.9) | 137.0  (72.7–258.5) | 0.34 (0.21–0.49) | 0.99* |  |
| Is the behaviour of the child, his or her carers and their interaction appropriate? | No | 85 (0.5) | 11 (20.0) | 65.3  (32.3–131.9) | 0.21 (0.11–0.35) | 0.99* |  |
| Are findings of the head-to-toe examination in accordance with the history? | No | 54 (0.3) | 9 (16.4) | 82.1  (37.9–178.2) | 0.17 (0.08–0.30) | 0.99* |  |
| Are there other signals that make you doubt the safety of the child or other family members? | Yes | 170 (0.9) | 30 (54.5) | 182.9  (102.3–327.4) | 0.59 (0.44–0.72) | 0.99* |  |
| ≥1 question positive |  | 420 (2.3) | 44 (80.0) | 189.8  (97.3–370.4) | 0.8 (0.67–0.89) | 0.98* |  |

*P-values for all items <0.001. Unknown outcome of screened negatives are analysed as negative outcome.*

** Maximum range of 95% CI 0.98–0.994*

**4C: Univariate analysis of questions from UMC dataset (SPUTOVAMO)**

| **Univariate analysis of questions from UMC dataset (SPUTOVAMO)** | | | | | | |
| --- | --- | --- | --- | --- | --- | --- |
| Items | Positive answers | No. of positive answers in total population n=4290 (%) | No. of positive answers among cases of suspected abuse (physical abuse/neglect)  n=9 (%) | OR (95% CI) | Sensitivity (95% CI) | Specificity (95% CI) |
| Injury compatible with history and corresponding to age of child? | No | 65 (1.5) | 4 (44.4) | 55.3  (14.5–211.13) | 0.44 (0.14–0.79) | 0.99* |
| History consistent when repeated? | No | 19 (0.4) | 1 (11.1) | 29.6  (3.5–249.1) | 0.11 (0.003–0.48) | 0.99* |
| Delay in ER attendance without satisfactory explanation? | Yes | 40 (0.9) | 1 (11.1) | 13.6  (1.7–111.3) | 0.11 (0.003–0.48) | 0.99* |
| Father/mother and child: appropriate behaviour/interaction? | No | 21 (0.5) | 5 (55.6) | 333.2  (81.9–1355.9) | 0.56 (0.21–0.86) | 0.99* |
| Top-to-toe examination: suspect? | Yes | 31 (0.7) | 3 (33.3) | 75.9  (18.1–318.9) | 0.33 (0.07–0.70) | 0.99* |
| ≥1 positive question |  | 126 (2.9) | 8 (88.9) | 282.2  (35.0–2274.9) | 0.89 (0.52–0.99) | 0.97 (0.97-0.98) |

*P-values for all items <0.001. Unknown outcome of screened negatives are analysed as negative outcome.*

** Maximum range of 95% CI 0.98–0.99*

**Online Resource 5: Screening questions after imputation**

| Harmonized screening questions | | Dataset AMC  n=3,136 (%) | Dataset EMC  n=18,159 (%) | Dataset UMC  n=3,668 (%) | Total dataset  n=24,963 (%) |
| --- | --- | --- | --- | --- | --- |
| Question 1: Injury compatible with history and corresponding to child’s developmental level | | | | | |
|  | Yes | 2,947.8 (94.0) | 18,073.2 (99.5) | 3,606 (98.3) | 24,627 (98.7) |
|  | No* | 188.2 (6.0) | 85.8 (0.5) | 62 (1.7) | 336 (1.3) |
| Question 2: Unnecessary delay in seeking medical help | | | | | |
|  | Yes* | 80.5 (2.6) | 138.3 (0.8) | 40 (1.1) | 258.8 (1.0) |
|  | No | 3,055.5 (97.4) | 18,020.7 (99.2) | 3,628 (98.9) | 24,704.2 (99.0) |
| Question 3: Consistent history | | | | | |
|  | Yes | 3,119.1 (99.5) | 18,080.9 (99.6) | 3,649 (99.5) | 24,849 (99.5) |
|  | No* | 16.9 (0.5) | 78.1 (0.4) | 19 (0.5) | 114 (0.5) |
| Question 4: Appropriate behaviour of the child, the parents and appropriate interaction | | | | | |
|  | Yes | 3,076 (98.1) | 18,072.4 (99.5) | 3,647 (99.4) | 24,795.4 (99.3) |
|  | No* | 60 (1,9) | 86.6 (0.5) | 21 (0.6) | 167.6 (0.7) |
| Question 5: Physical injuries found with top-toe examination suspect for child maltreatment | | | | | |
|  | Yes* | 69 (2.2) | 52.3 (0.3) | 35.5 (1.0) | 156.8 (0.6) |
|  | No | 3,067 (97.8) | 18,106.7 (99.7) | 3,632.5 (99.0) | 24,806.2 (99.4) |
| Question 6: Other signals that make you doubt the safety of child/family | | | | | |
|  | Yes* | n.a. | 163 (0.9) | n.a. | n.a. |
|  | No | n.a. | 17,878 (98.5) | n.a. | n.a. |

**positive answer on screening question = positive result. Multiple imputation* *included the following constraints: dataset, hospital (n=8), gender, age, consensus diagnosis (outcome) and all harmonized questions.*

*n.a. = not applicable*

**Online Resource 6: Sensitivity analysis for missing values on screening questions (outcome measure A)**

| **Sensitivity analysis reduced model** | | aOR (95% CI) | p-value |
| --- | --- | --- | --- |
| (Constant)^*^ |  | 0.007 | 0.98 |
| Question 1^*^ | Injury compatible with history and corresponding to child’s developmental level | 10.75 (6.21–18.61) | <0.001 |
| Question 2^*^ | Unnecessary delay in seeking medical help | 3.43 (1.71–6.87) | <0.001 |
| Question 4^*^ | Appropriate behaviour of the child, the parents and appropriate interaction | 15.55 (8.66–27.90) | <0.001 |

*Model is adjusted for age, gender, hospital (n=8) and screening questions. Unknown outcome of screened negatives are analysed as negative outcome (outcome measure A). Discriminative value: AUC 0.78 (95% CI 0.72–0.84)*

**Online Resource 7: Calibration plot: observed proportion vs. predicted probability of the screening instrument for 3 internal-external cross-validations (outcome measure B)**


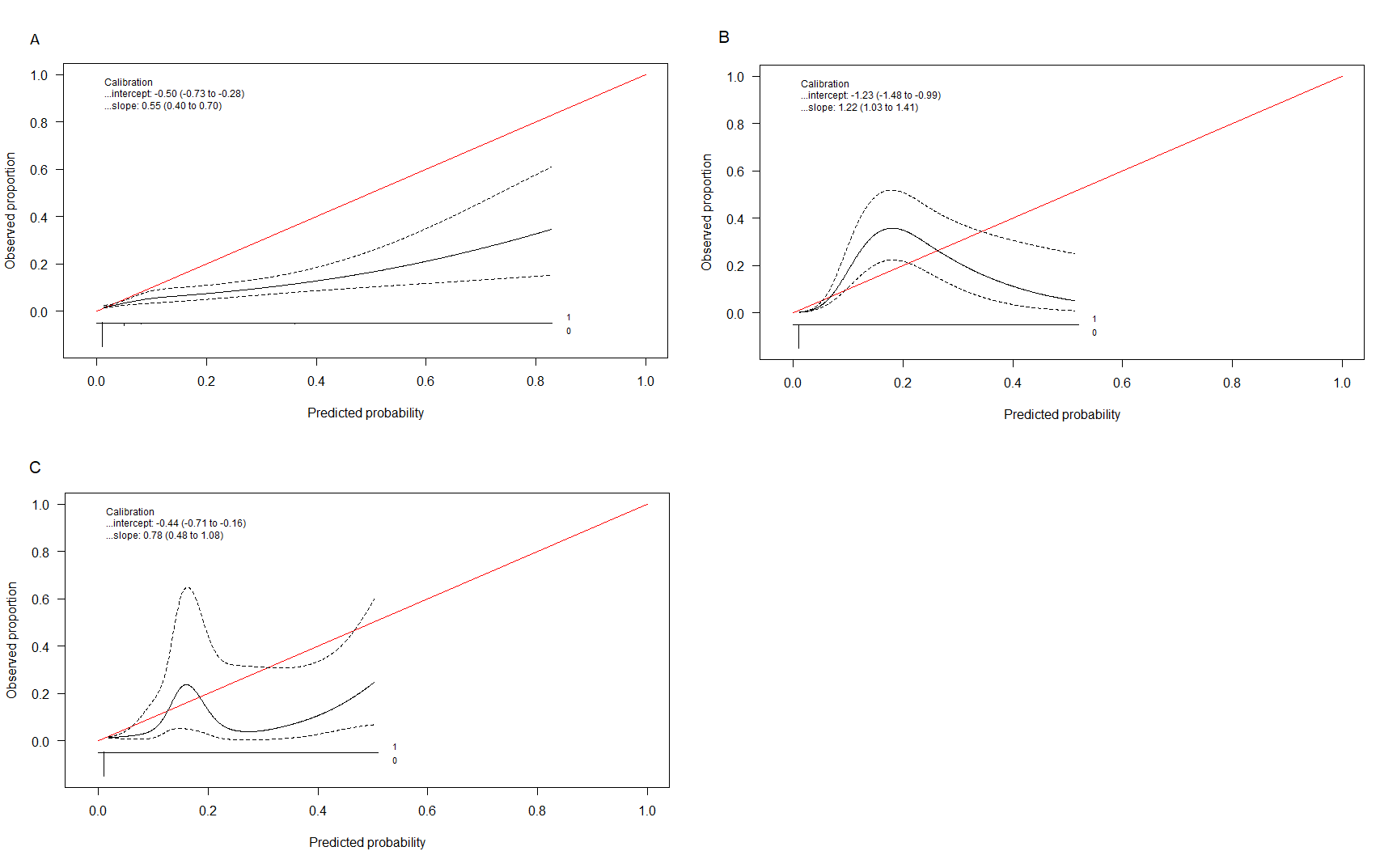


*The solid red line with a slope of 1 and intercept of 0 represents ideal prediction accuracy. The dotted lines indicate the 95% confidence interval. Unknown outcomes for the negative-screened cases were supplemented based on the reports to the Safe at Home centre (outcome measure B).*

*A. Model developed on leave-out AMC dataset, validated on AMC dataset (n=3,316)*

*B. Model developed on leave-out EMC dataset, validated on EMC dataset (n=18,159)*

*C. Model developed on leave-out UMC dataset, validated on UMC dataset (n=3,668)*

**Online Resource 8: Validity of screening instruments for the recognition of (suspected) child maltreatment at the emergency department**

*PPV = positive predictive value, NPV = negative predictive value*

*Numbers 1 and 2 are the ESCAPE instrument [1, 2]; 3 is SPUTOVAMO* [3]; 4 is SPUTOVAMO & TTI* [3]; 5 is SPUTOVAMO-R2 [4]; 6 is SPUTOVAMO-R3 [4]; 7 is SPUTOVAMO-R [5].*

**These estimates are an approach of the actual values, because of differential verification methods being used to verify positives and negatives*

**References Online Resource 8**

1. Louwers EC, Korfage IJ, Affourtit MJ, Ruige M, van den Elzen AP, de Koning HJ, et al. Accuracy of a screening instrument to identify potential child abuse in emergency departments. Child Abuse Negl. 2014;38(7):1275-81. <https://doi.org/10.1016/j.chiabu.2013.11.005>.

2. Dinpanah H, Akbarzadeh Pasha A. Potential Child Abuse Screening in Emergency Department; a Diagnostic Accuracy Study. Emerg (Tehran). 2017;5(1):e8.

3. Teeuw AH, Kraan RBJ, van Rijn RR, Bossuyt PMM, Heymans HSA. Screening for child abuse using a checklist and physical examinations in the emergency department led to the detection of more cases. Acta Paediatr. 2019;108(2):300-13. <https://doi.org/10.1111/apa.14495>.

4. Schouten MCM, van Stel HF, Verheij TJM, Houben ML, Russel IMB, Nieuwenhuis EES, et al. The Value of a Checklist for Child Abuse in Out-of-Hours Primary Care: To Screen or Not to Screen. PLoS One. 2017;12(1):e0165641. <https://doi.org/10.1371/journal.pone.0165641>.

5. Sittig JS, Uiterwaal CS, Moons KG, Russel IM, Nievelstein RA, Nieuwenhuis EE, et al. Value of systematic detection of physical child abuse at emergency rooms: a cross-sectional diagnostic accuracy study. BMJ Open. 2016;6(3):e010788. <https://doi.org/10.1136/bmjopen-2015-010788>.
